# Archaeal community variation in the Qinhuangdao coastal aquaculture zone revealed by high-throughput sequencing

**DOI:** 10.1371/journal.pone.0218611

**Published:** 2019-06-21

**Authors:** Shuping Wang, Xin Zheng, Huijuan Xia, Di Shi, Juntao Fan, Pengyuan Wang, Zhenguang Yan

**Affiliations:** State Key Laboratory of Environmental Criteria and Risk Assessment, Chinese Research Academy of Environmental Sciences, Beijing, China; CAS, CHINA

## Abstract

The differences in archaeal diversity and community composition in the sediments and waters of the Qinhuangdao coastal aquaculture zone were investigated. Furthermore, the associations between dominant archaeal taxa with geographic and environmental variables were evaluated. High-throughput sequencing of archaeal 16S rRNA genes yielded a total of 176,211 quality-filtered reads and 1,178 operational taxonomic units (OTUs) overall. The most abundant phylum and class among all communities were Thaumarchaeota and Nitrososphaeria, respectively. Beta diversity analysis indicated that community composition was divided into two groups according to the habitat type (i.e., sediments or waters). Only 9.8% OTUs were shared by communities from the two habitats, while 73.9% and 16.3% of the OTUs were unique to sediment or water communities, respectively. Furthermore, the relative abundances of the dominant OTUs differed with habitat type. Investigations of relationships between dominant OTUs and environmental variables indicated that some dominant OTUs were more sensitive to variation in environmental factors, which could be due to individual taxonomic differences in lifestyles and biological processes. Overall, the investigation of archaeal community variation within the Qinhuangdao coastal aquaculture zone provides an important baseline understanding of the microbial ecology in this important ecosystem.

## Introduction

The Bohai Sea has become one of the most polluted marine systems in China, and its ecosystem is rapidly degrading [1]. The city of Qinhuangdao is on the coast of the Bohai Sea and is well known for its seaside scenery and offshore mariculture industry [2,3]. The dominant mariculture practice in the Qinhuangdao coastal area is raft cultivation, with Bay scallops (*Argopecten irradians*) as the primary cultivated shellfish species. *A*. *irradians* farming has been conducted in Qinhuangdao for over 30 years, more than that, the scale of aquaculture expanded quickly after 2000 and now accounts for more than 70% of its production in China [4,5]. Seabeds below *A*. *irradians’* farms are usually enriched in organic materials, and these benthic environments can exhibit pronounced variation in sediment geochemistry and benthic community structures [5]. Several studies have investigated the water quality, phytoplankton communities, and bacterial communities in Qinhuangdao coastal areas. However, similar studies have yet to be conducted for the Qinhuangdao coastal aquaculture zone [6–10]. A complete understanding of microbial diversity and abundances of aquaculture zones is crucial to understanding these ecosystems. Nevertheless, there is a lack of available data for microbial communities in the ecosystems of the Qinhuangdao coastal aquaculture zone.

Microbial communities and their associated metabolic activities in marine waters and sediments have profound impacts on global biogeochemical cycles, including those for nitrogen, carbon, and sulfur, in addition to impacting food webs [11,12]. Archaeal populations are typically considered to thrive in extreme environments, but represent small fractions of the total microbial communities in marine systems and are thus considered part of the rare biosphere in marine ecosystems [13]. Regardless, marine archaeal populations significantly impact global biogeochemical cycles and greenhouse gas emissions [14]. For example, ANME-1 and ANME-2 archaea in marine systems perform anaerobic oxidation of methane [15]. The oxidation of ammonia to nitrite can be performed by the phylum Thaumarchaeota whcih possess the ammonia monooxygenase subunit A (*amoA*) genes [16–18]. Therefore, understanding variation of archaeal communities in aquaculture environments is crucial for predicting biogeochemical fluxes in such environments.

The number of archaeal species known from environmental 16S rRNA gene sequences has far surpassed that of cultured archaea. Consequently, 16S rRNA gene high-throughput sequencing has been used to investigate the distribution of Archaea among various environments [19–21]. Investigations of microbial diversity in the Qinhuangdao coastal ecosystem have mainly focused on evaluating bacterial communities in waters and intertidal sediments [7,8,10]. In contrast, nothing is known of the archaeal community composition and diversity in Qinhuangdao coastal aquaculture environments.

Organic matter in sediments is 10^4^–10^5^ fold higher than in waters and serves as an important energy source for microorganisms [13]. Indeed, the relative abundances of microbial taxa can be shaped by variation in organic carbon availability and mineralogy [22]. Archaea account for more than 20% of the bacterial and archaeal communities of ocean waters and dominate microbial communities in sediments [23,24]. The sediments and their corresponding benthic waters are heterogeneous and can also drive variation in microbial community composition. Specifically, geographic and environmental factors can exert selective pressures on the microbial communities. For example, adaptive shifts in bacterioplankton community composition and species interactions occurred in response to nutrient pollution in highly polluted water bodies [25]. Therefore, connecting archaeal distributions with habitat types and environmental variables will promote a better understanding of archaeal metabolic functions and biogeochemical processes within Bohai coastal aquaculture environments.

In this study, archaeal communities in waters and sediments were investigated at four stations in the Bohai coastal aquaculture zone using high-throughput Illumina sequencing of community 16S rRNA genes. The associations of archaeal taxa with habitat types in addition to geographic and environmental parameters were investigated. The main objectives of this study were to (1) compare differences in diversity among archaeal communities within sediments and waters of the aquaculture zone and (2) evaluate whether geographical or environmental variables influence the relative abundances of dominant archaeal taxa in the coastal waters. This study represents the first report of archaeal diversity, community composition, and the role of external factors influencing archaeal diversity within ecosystems of the Qinhuangdao coastal aquaculture zone.

## Materials and methods

### Site description, sample collection, and physicochemical analyses

**Fig 1:**
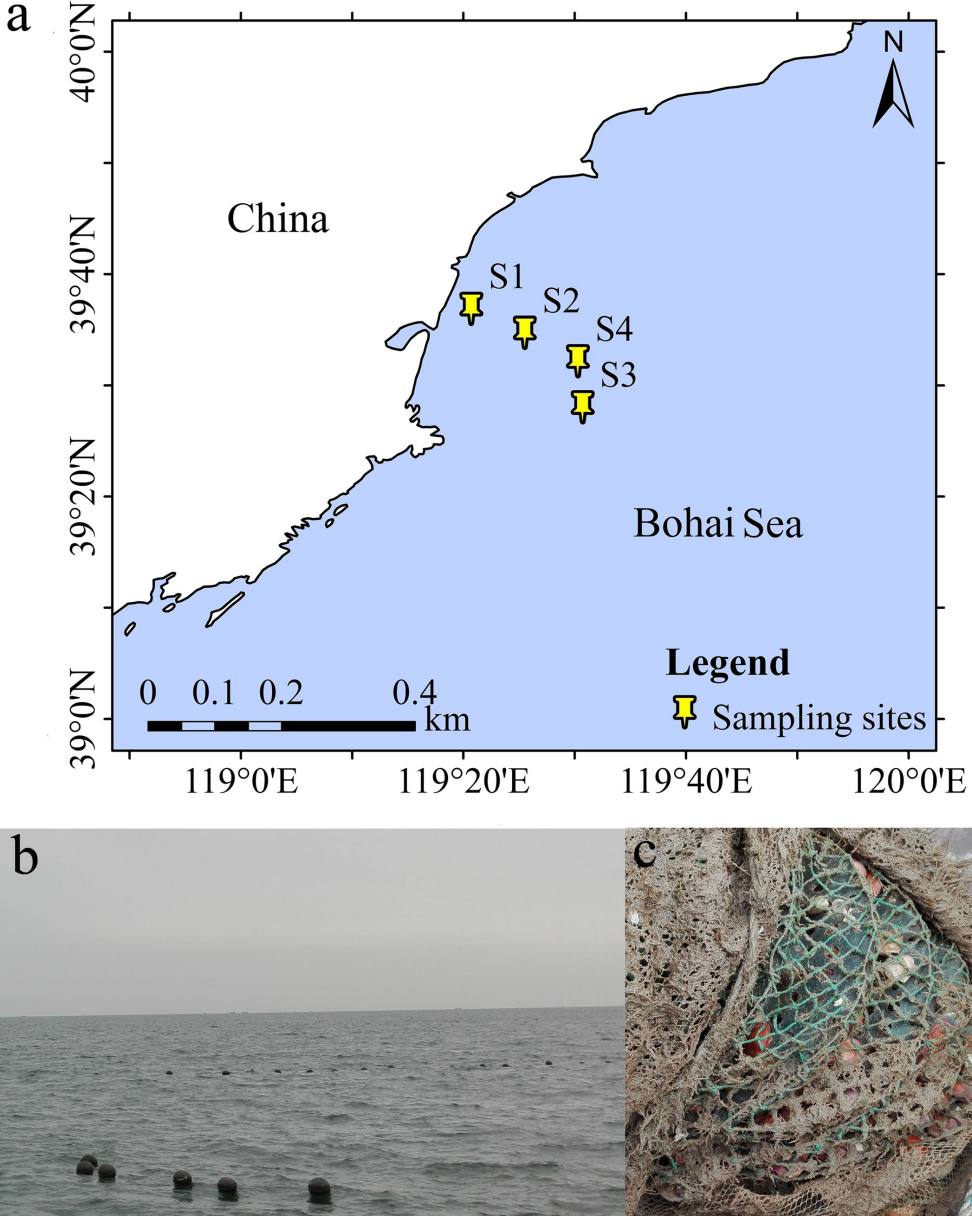
(a) A map showing sampling locations within the Qinhuangdao coastal aquaculture zone. ArcGIS 10.1 software (http://www.esri.com/software/arcgis) was used to develop the map. (b) Raft cultivation of *A*. *irradians* in the Qinhuangdao coastal aquaculture zone. (c) Mesh cage used for cultivation.

Raft cultures for scallop cultivation are primarily used in the study area and the farmed scallop primarily originate from the lower layer seawater. Ocean waters were collected on July 26, 2017 from four different sites that were located in the *A*. *irradias* farming area of the bay (Fig 1). S1 waters (39°36’53” N, 119°20’43” E) came from 8 m depth, S2 (39°34’43” N, 119°25’32” E) from 10 m depth, S3 (39°28’2” N, 119°30’43” E) from 13 m depth, and S4 (39°32’10” N, 119°30’18” E) from 15 m depth, which was the deepest point in the water column. In addition, benthic sediments were concomitantly collected at the S1 (39°36’53” N, 119°20’43” E) and S2 (39°34’43” N, 119°25’32” E) sites. Ten litres of seawater were collected in triplicate at each location, and the triplicate samples were homogeneously mixed prior to filtration. Five litres of pooled water from each site were filtered through a 0.2-μm filter membrane (Millipore, Billerica, USA) and the filter membranes were then stored at -80°C until further analysis. Sediments were collected using a stainless steel static gravity corer (UWITEC, Mondsee, Austria). Five grams of surface sediments (0–5 cm) were carefully removed with a stainless steel spoon and stored in sterile 5 ml storage tubes. Samples were immediately preserved on dry ice after sampling, and then transported to the laboratory. Several water physicochemical parameters were measured with a portable YSI Pro Plus Multiparameter instrument (YSI, Yellow Springs, OH, USA) including water temperature, dissolved oxygen (DO), salinity, turbidity, electrical conductivity (EC), and total dissolved solids (TDS). Total nitrogen (TN), total phosphorus (TP), and total organic carbon (TOC) were also measured in water samples using the protocols described in “Specification for oceanographic survey” (GB/T 12763.4–2007).

### DNA isolation, PCR amplification, and high-throughput sequencing of 16S rRNA genes

Total community DNA was isolated directly from membrane samples using an E.Z.N.A.^TM^ water DNA kit (Omega Bio-Tek Inc., USA), according to the manufacturer’s protocols using an integrated mechanical and chemical extraction procedure. DNA was also extracted from 1 g of sediments using a PowerMax Soil DNA isolation Kit (12988–10, MOBIO Laboratories, Inc, Carlsbad, CA). Purified DNA was dissolved in 50 μl of ddH_2_O and stored at -20°C. DNA quantity and quality were determined using agarose gel electrophoresis and spectrophotometric quantification using a NanoDrop ND 2000 instrument (Thermo Fisher Scientific, Waltham, MA, USA).

The hypervariable V4+V5 sequence region of archaeal 16S rRNA genes were amplified using the universal primers Arch519F (5’-CAGCCGCCGCGGTAA-3’) and Arch915R (5’-GTGCTCCCCCGCCAATTCCT-3’) [26]. PCR amplification was conducted as previously described [27]. DNA sequencing of PCR amplicons was then performed on the Illumina Miseq platform using paired-end 250 bp sequencing and a V3 Miseq Reagent Kit at the Personal Biotechnology Company (Novogene, Tianjin, China).

### Data processing

Raw sequence reads were quality filtered in QIIME [28] using stringent specifications. Paired reads were merged using FLASH-1.2.8 [29]. Operational taxonomic units (OTUs) were then defined at the 97% nucleotide identity threshold with UCLUST, as implemented in QIIME [30]. Representative sequences from each OTU were then taxonomically classified with BLAST [31] searches against the SILVA v132 reference database using QIIME [32]. Singleton OTUs with only a single read were removed from the analysis. Archaeal community alpha diversity was then estimated using the Shannon diversity index. Community compositional similarity was also estimated using the Bray-Curtis distance metric. The alpha- and beta-diversity indices were both calculated using QIIME. In addition, rarefaction curves were generated using UPARSE to quantify the level of diversity that was captured with the sequencing efforts.

### Statistical analyses

The relative abundances of each taxonomic group were determined for each community, while excluding sequences annotated as Bacteria, Eukarya, and unclassifiable groups. The 16S rRNA gene sequences of the 50 most abundant archaeal OTUs were aligned using Clustal X [33]. A phylogenetic tree was then obtained from the alignment using maximum likelihood methods and 1,000 bootstrap replicates in the MEGA 6 software package [34]. Dominant OTUs were defined as the 50 most relatively abundant OTUs. Venn diagrams of OTU members shared among samples were constructed with an online tool (http://www.omicshare.com/tools/Home/Soft/venn). Heatmaps were also constructed to visualize among-sample diversity using R software packages (http://cran.r-project.org/, version 3.2.2). Spearman correlational analysis was conducted using the SPSS 17.0 software program (Chicago, IL, USA), with two-tailed *p* values less than 0.01 considered as statistically significant.

### Nucleotide sequence accession numbers

16S rRNA gene sequences generated in this study are deposited in GenBank under the accession number PRJNA508582.

## Results

### Rarefaction curve analysis and taxonomic classifications

**Fig 2:**
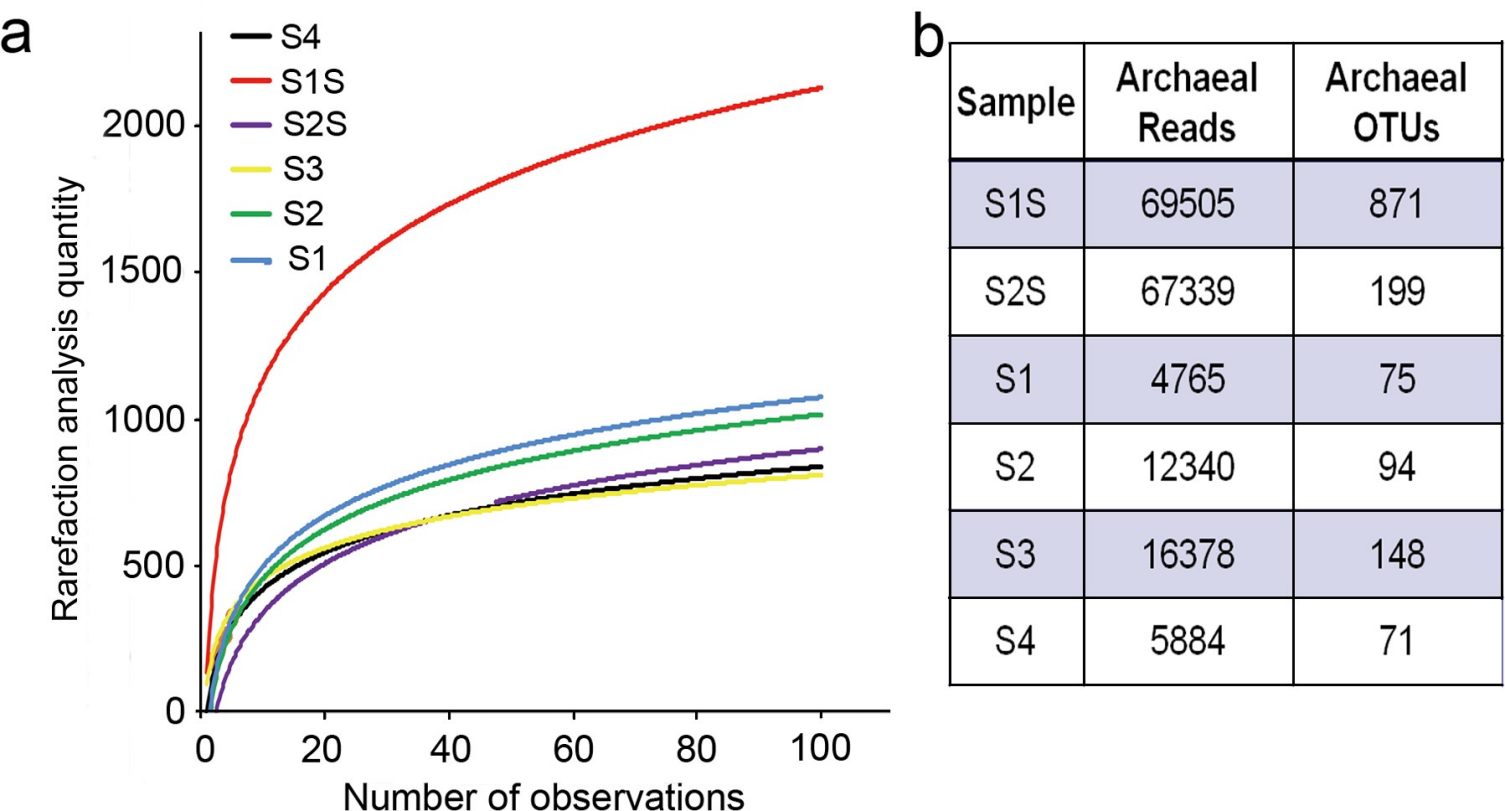
16S rRNA gene sequence diversity summary and taxonomic assignments. (a) Rarefaction curves of OTU richness for archaeal communities. (b) Information for archaeal sequences from different samples.

A total of 447,217 16S rRNA gene sequences (74,536 ± 12,591 reads [mean ± standard deviation]) were obtained from sediment and water samples after quality-filtering raw read sequences. A total of 1,178 OTUs were observed overall among the archaeal communities. Rarefaction curves for each sample reached asymptotes, indicating that native archaeal diversity was well covered with sufficient sequencing depth (Fig 2A). A total of 871, 199, 75, 94, 148, and 71 OTUs were observed in the S1 sediment, S2 sediment and the S1, S2, S3, and S4 water samples, respectively (Fig 2B).

### Community compositional variation

**Fig 3:**
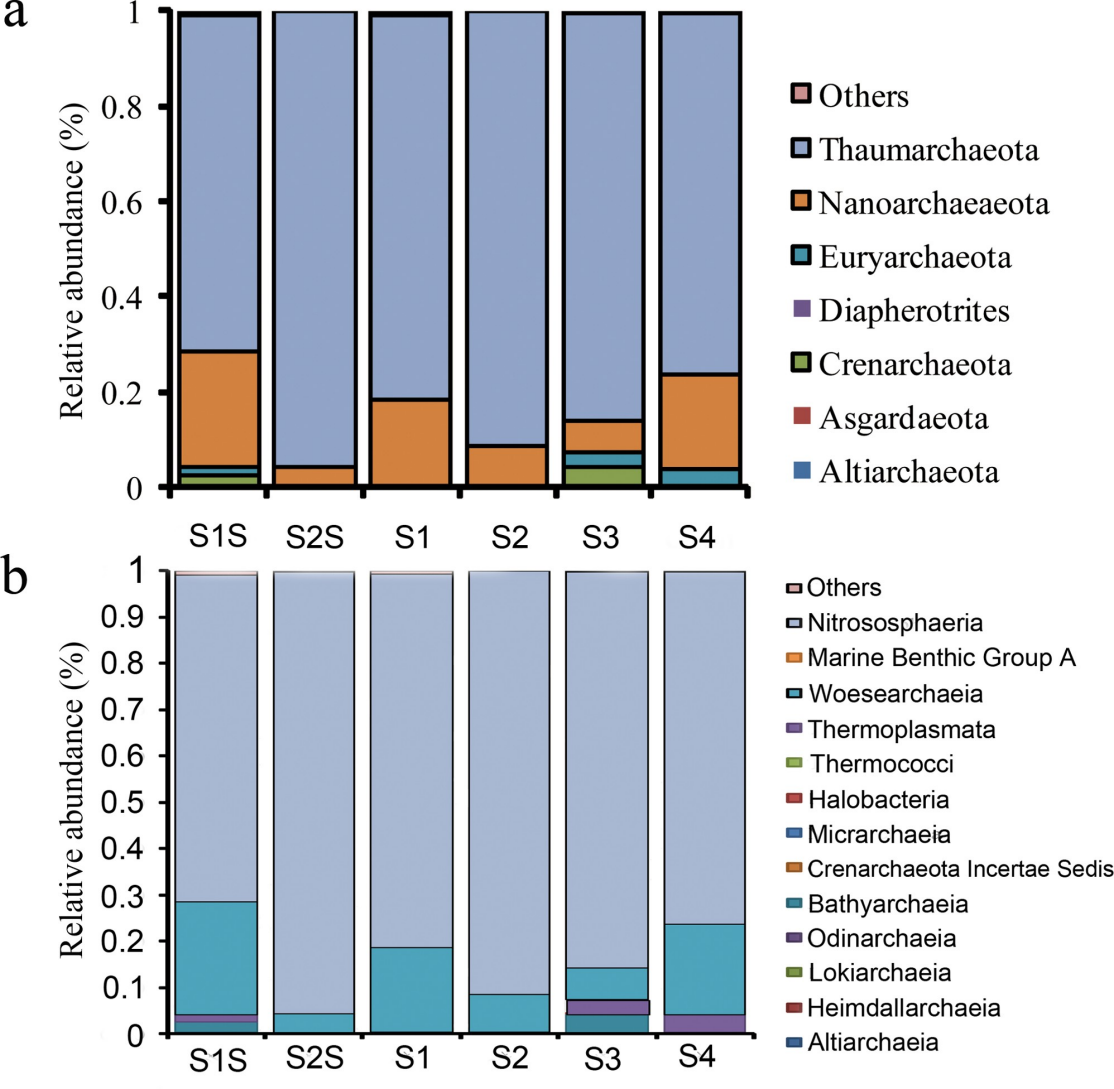
Taxonomic profiles of archaeal communities at the (a) phylum and (b) class levels. Taxonomic groups with low abundances are indicated as “others”.

To investigate the taxonomic composition of archaeal communities, OTUs were classified at the phylum- and class levels (Fig 3). OTUs that could not be assigned to any phylum or class are indicated as ‘others’. Seven major archaeal phyla were identified in the sediment and water communities, including Thaumarchaeota, Nanoarchaeaeota, Crenarchaeota, Euryarchaeota, Asgardaeota, Altiarchaeota, and Diapherotrites (Fig 3A). A majority of the archaeal 16S rRNA gene sequences were associated with the Thaumarchaeota and Nanoarchaeaeota phyla, which represented 70.7%-95.6% and 4.1%-24.3% of the total sequences, respectively. The remaining six phyla (inclusive of ‘others’) accounted for only 2.7%-4.2% of the communities overall. At the class level, OTUs comprised 13 archaeal classes, with the most abundant corresponding to Nitrososphaeria (70.7%-95.6%). Woesearchaeia were also abundant in all of the samples, representing 4.1%–24.3% of the community compositions (Fig 3B).The Altiarchaeota and Diapherotrites phyla were only present as minor archaeal community members. In addition, the Altiarchaeota and Diapherotrites were only found in the sediment communities (Fig 3A).

### Archaeal diversity differs between sediments and waters

**Fig 4:**
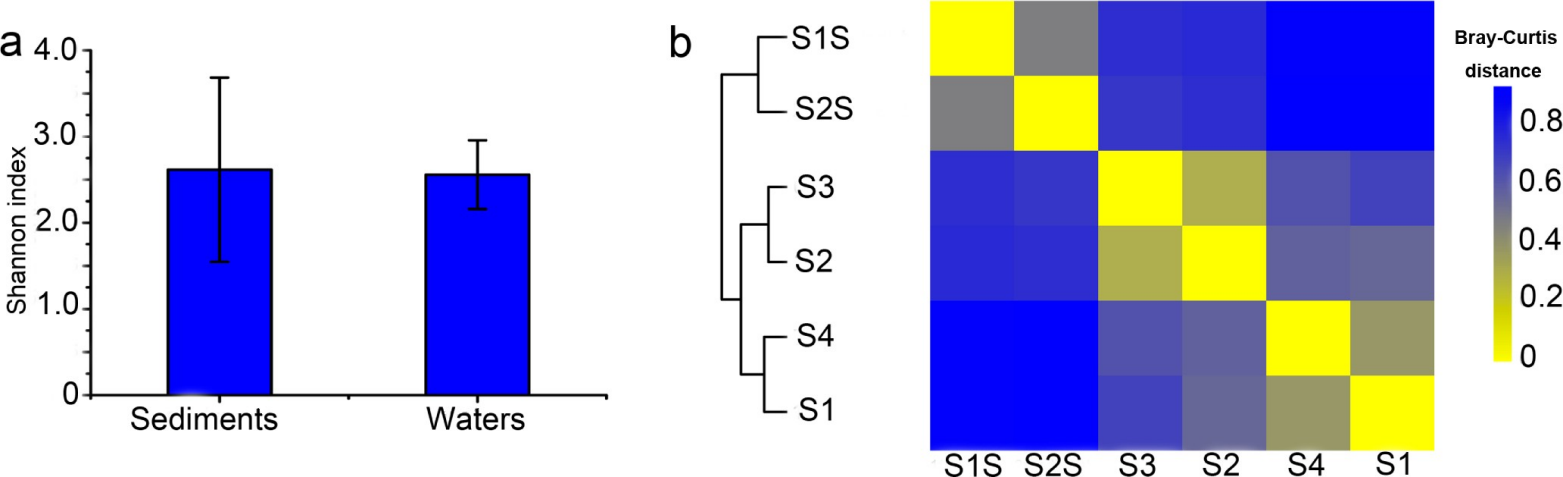
Summary of archaeal community variation between sediments and waters. (a) Shannon diversity index values for archaeal communities. (b) Comparison of archaeal community compositions using Bray-Curtis distances.

Shannon indices of sediment communities were slightly higher than those of waters, but were not significantly different (*p* > 0.05; Fig 4A). Cluster analysis of beta diversity indicated that the archaeal communities of sediments were significantly different than those of the water communities (Fig 4B).Taken together, these results indicate that the archaeal communities differed between sediments and waters.

### Shared and unique OTUs between sediments and waters

**Fig 5:**
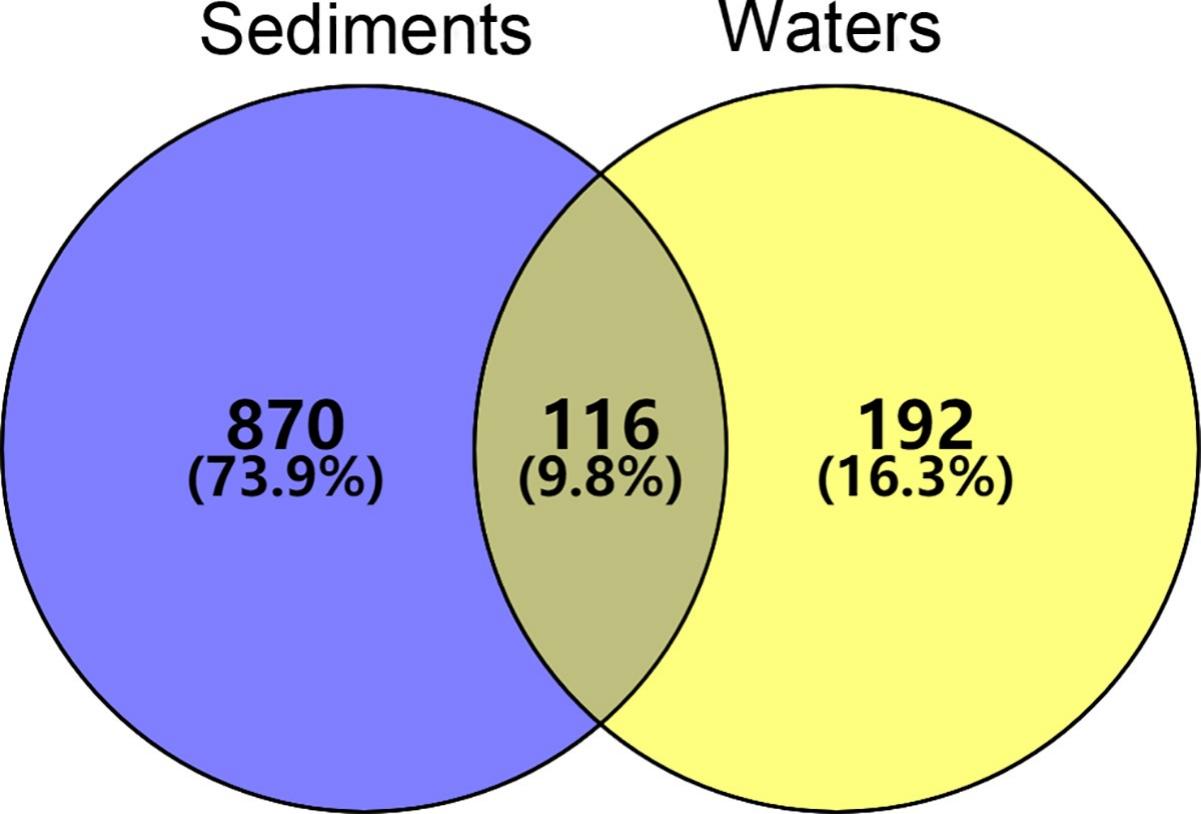
Venn diagrams showing the distribution of unique and shared OTUs between habitats. The numbers and relative proportions of OTUs in each sample are indicated by their respective circles.

A total of 116 OTUs were shared between sediment and water communities, while 870 and 192 OTUs were unique to each of the habitats, respectively (Fig 5). Most of the dominant OTUs in either habitat were shared amongst both habitats. For example, OTU932, OTU3223, and OTU371, which were all classified as Candidatus *Nitrosopumilus*, were the most abundant shared OTUs. OTU3191, which was also classified as Candidatus *Nitrosopumilus*, was the most abundant OTU in sediments not observed in water samples. Conversely, OTU67 (classified as Nitrososphaeraceae), OTU2438 (Candidatus *Nitrosopumilus*), and OTU326 (Marine Group II) were the three most abundant OTUs that were only found in water communities (Table 1).

**Table 1:** The 50 most abundant OTUs and their taxonomic affiliations.

Feature ID	Taxon
**OTU932**	Thaumarchaeota;Nitrososphaeria;Nitrosopumilales;Nitrosopumilaceae;Candidatus *Nitrosopumilus*
**OTU3223**	Thaumarchaeota;Nitrososphaeria;Nitrosopumilales;Nitrosopumilaceae;Candidatus *Nitrosopumilus*
**OTU371**	Thaumarchaeota;Nitrososphaeria;Nitrosopumilales;Nitrosopumilaceae;Candidatus *Nitrosopumilus*
**OTU1762**	Thaumarchaeota;Nitrososphaeria;Nitrosopumilales;Nitrosopumilaceae;Candidatus *Nitrosopumilus*
**OTU1930**	Thaumarchaeota;Nitrososphaeria;Nitrosopumilales;Nitrosopumilaceae
**OTU829**	Thaumarchaeota;Nitrososphaeria;Nitrosopumilales;Nitrosopumilaceae;Candidatus *Nitrosopumilus*
**OTU249**	Thaumarchaeota;Nitrososphaeria;Nitrosopumilales;Nitrosopumilaceae;Candidatus *Nitrosopumilus*
**OTU2466**	Thaumarchaeota;Nitrososphaeria;Nitrosopumilales;Nitrosopumilaceae;Candidatus *Nitrosopumilus*
**OTU281**	Thaumarchaeota;Nitrososphaeria;Nitrosopumilales;Nitrosopumilaceae
**OTU1250**	Thaumarchaeota;Nitrososphaeria;Nitrosopumilales;Nitrosopumilaceae
**OTU3559**	Thaumarchaeota;Nitrososphaeria;Nitrosopumilales;Nitrosopumilaceae;Candidatus *Nitrosopelagicus*;uncultured archaeon
**OTU3455**	Thaumarchaeota;Nitrososphaeria;Nitrosopumilales;Nitrosopumilaceae
**OTU1356**	Thaumarchaeota;Nitrososphaeria;Nitrosopumilales;Nitrosopumilaceae;Candidatus *Nitrosopelagicus*;uncultured archaeon
**OTU984**	Thaumarchaeota;Nitrososphaeria;Nitrosopumilales;Nitrosopumilaceae;Candidatus *Nitrosopumilus*
**OTU1887**	Thaumarchaeota;Nitrososphaeria;Nitrosopumilales;Nitrosopumilaceae;Candidatus *Nitrosopumilus*;uncultured archaeon
**OTU954**	Thaumarchaeota;Nitrososphaeria;Nitrosopumilales;Nitrosopumilaceae
**OTU3191**	Thaumarchaeota;Nitrososphaeria;Nitrosopumilales;Nitrosopumilaceae;Candidatus *Nitrosopumilus*
**OTU770**	Thaumarchaeota;Nitrososphaeria;Nitrosopumilales;Nitrosopumilaceae;Candidatus *Nitrosopelagicus*;uncultured archaeon
**OTU3181**	Thaumarchaeota;Nitrososphaeria;Nitrosopumilales;Nitrosopumilaceae;*Cenarchaeum*;uncultured archaeon
**OTU1400**	Thaumarchaeota;Nitrososphaeria;Nitrosopumilales;Nitrosopumilaceae;Candidatus *Nitrosopumilus*;uncultured archaeon
**OTU2462**	Nanoarchaeaeota;Woesearchaeia;uncultured bacterium
**OTU1071**	Thaumarchaeota;Nitrososphaeria;Nitrosopumilales;Nitrosopumilaceae;Candidatus *Nitrosopumilus*;uncultured archaeon
**OTU3289**	Thaumarchaeota;Nitrososphaeria;Nitrosopumilales;Nitrosopumilaceae
**OTU1158**	Nanoarchaeaeota;Woesearchaeia;uncultured bacterium
**OTU853**	Thaumarchaeota;Nitrososphaeria;Nitrosopumilales;Nitrosopumilaceae;Candidatus *Nitrosopumilus*
**OTU896**	Euryarchaeota;Thermoplasmata;Marine Group II;marine metagenome
**OTU1433**	Nanoarchaeaeota;Woesearchaeia;uncultured bacterium
**OTU927**	Thaumarchaeota;Nitrososphaeria;Nitrosopumilales;Nitrosopumilaceae;Candidatus *Nitrosopumilus*;uncultured archaeon
**OTU1592**	Thaumarchaeota;Nitrososphaeria;Nitrosopumilales;Nitrosopumilaceae
**OTU67**	Thaumarchaeota;Nitrososphaeria;Nitrososphaerales;Nitrososphaeraceae
**OTU2990**	Nanoarchaeaeota;Woesearchaeia
**OTU2132**	Crenarchaeota;Bathyarchaeia;uncultured crenarchaeote
**OTU2041**	Thaumarchaeota;Nitrososphaeria;Nitrosopumilales;Nitrosopumilaceae
**OTU3461**	Thaumarchaeota;Nitrososphaeria;Nitrosopumilales;Nitrosopumilaceae;Candidatus *Nitrosopelagicus*;uncultured archaeon
**OTU1810**	Nanoarchaeaeota;Woesearchaeia;uncultured bacterium
**OTU173**	Thaumarchaeota;Nitrososphaeria;Nitrosopumilales;Nitrosopumilaceae;Candidatus *Nitrosopumilus*
**OTU1077**	Thaumarchaeota;Nitrososphaeria;Nitrosopumilales;Nitrosopumilaceae;Candidatus *Nitrosopumilus*
**OTU90**	Nanoarchaeaeota;Woesearchaeia
**OTU3457**	Nanoarchaeaeota;Woesearchaeia
**OTU1103**	Thaumarchaeota;Nitrososphaeria;Nitrosopumilales;Nitrosopumilaceae;Candidatus *Nitrosopumilus*;uncultured archaeon
**OTU2907**	Thaumarchaeota;Nitrososphaeria;Nitrosopumilales;Nitrosopumilaceae;Candidatus *Nitrosopumilus*
**OTU353**	Crenarchaeota;Bathyarchaeia;uncultured *crenarchaeote*
**OTU2438**	Thaumarchaeota;Nitrososphaeria;Nitrosopumilales;Nitrosopumilaceae;Candidatus *Nitrosopumilus*
**OTU306**	Thaumarchaeota;Nitrososphaeria;Nitrosopumilales;Nitrosopumilaceae;Candidatus *Nitrosopumilus*
**OTU1453**	*Thaumarchaeota*;*Nitrososphaeria*;*Nitrosopumilales*;*Nitrosopumilaceae*;*Cenarchaeum*;uncultured archaeon
**OTU1189**	Nanoarchaeaeota;Woesearchaeia
**OTU2890**	Nanoarchaeaeota;Woesearchaeia;uncultured bacterium
**OTU3503**	Thaumarchaeota;Nitrososphaeria;Nitrososphaerales;Nitrososphaeraceae
**OTU886**	Thaumarchaeota;Nitrososphaeria;Nitrosopumilales;Nitrosopumilaceae
**OTU1345**	Euryarchaeota;Thermoplasmata;SG8-5;uncultured archaeon

### Turnover of dominant OTUs is associated with different habitat types

**Fig 6:**
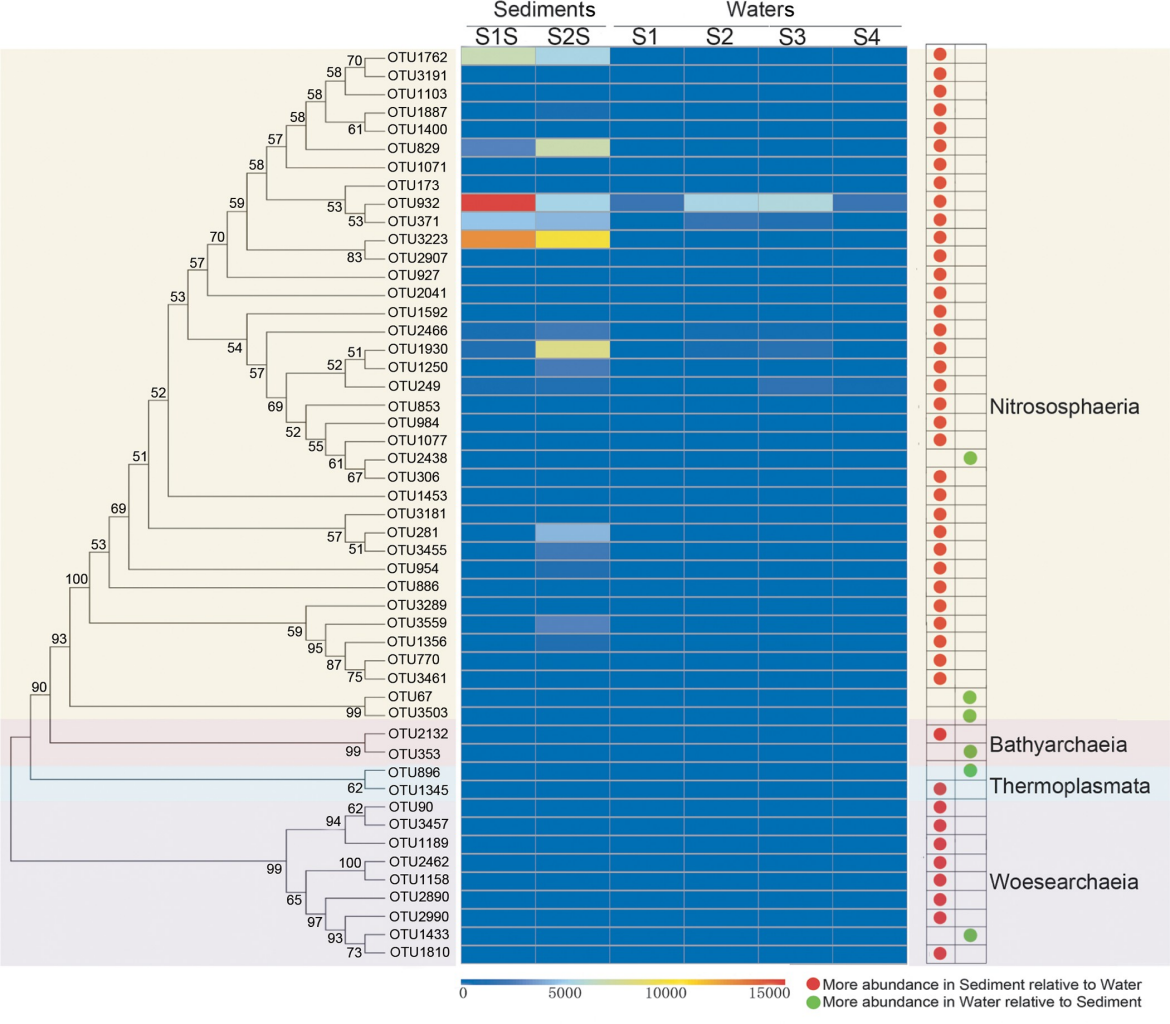
Maximum likelihood phylogenetic tree and distribution of dominant archaeal OTUs in marine sediments and waters of the Qinhuangdao coastal aquaculture zone. Only bootstrap values greater than 50% out of 1,000 replicates are shown. The relative abundances of OTUs are colored according to the corresponding heatmap legends.

A phylogenetic analysis was conducted using aligned 16S rRNA gene sequences from the 50 most abundant OTUs (Fig 6). The phylogenetic analysis indicated that these 50 OTUs could be categorized into four groups corresponding to the classes Nitrososphaeria, Bathyarchaeia, Thermoplasmata, and Woesearchaeia. The distributions of the relative abundances of these OTUs among communities were also explored. Most OTUs, and especially OTU1762, OTU829, OTU932, OTU371, OTU3223, OTU1930, OTU281, and OTU3559, were more abundant in sediments than in waters (Fig 6). In contrast, only six OTUs including OTU2438, OTU67, OTU3503, OTU353, OTU896, and OTU1433 were more abundant in water communities than in those of sediments.

### Associations between dominant OTUs with geographic or environmental variables

**Fig 7:**
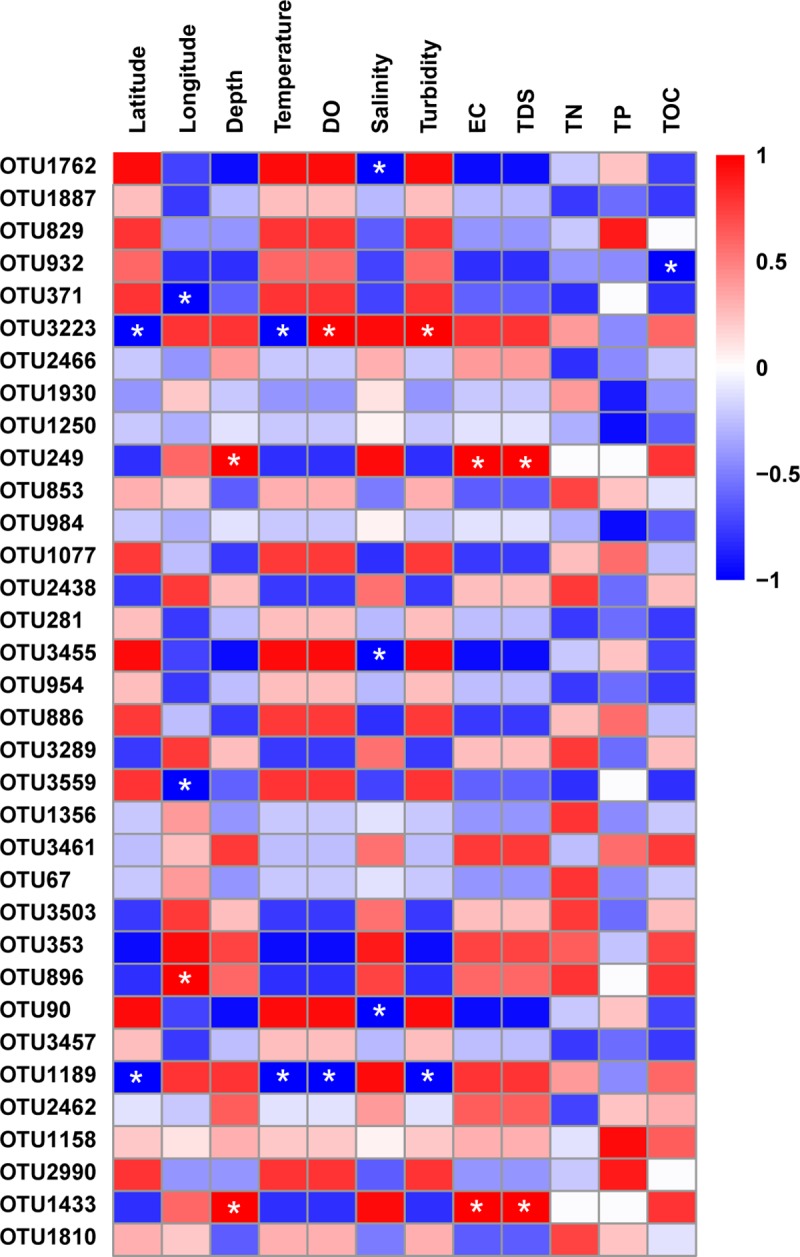
Spearman’s correlations between the abundances of dominant OTUs with geographic and environmental parameters. Correlation coefficient values are colored according to their magnitude as indicated by the heatmap legends. Only taxa that were significantly correlated with one of the parameters are shown. * indicates statistically significant values (*p* ≤ 0.01).

Taxonomic responses to varying geographic or environmental factors commonly differ. Archaeal OTU richness and Shannon diversity index values for the Bohai bay communities did not correlate with measured geographic or environmental variables (*p* > 0.05). Spearman correlation analyses were used to further explore the relationships between the abundances of dominant OTUs with geographic and environmental parameters (Fig 7). Most of the dominant archaeal OTUs were not significantly associated with any of the geographic or environmental variables that were measured. Nevertheless, the abundances of eleven dominant archaeal OTUs variably correlated with latitude, longitude, depth, temperature, DO, salinity, turbidity, EC, TDS, or TOC. Specifically, the abundances of OTU3223 (Candidatus *Nitrosopumilus*) and OTU1189 (Woesearchaeia) were negatively associated with latitude (*p* < 0.01). The relative abundances of OTU371 (Candidatus *Nitrosopumilus*) and OTU3559 (Candidatus *Nitrosopelagicus*) were significantly and negatively correlated with longitude (*p* < 0.01), while those of OTU896 (Marine Group II) were strongly and positively correlated with longitude (*p* < 0.01). In addition, the abundances of OTU249 (Candidatus *Nitrosopumilus*) and OTU1433 (Woesearchaeia) were significantly and positively correlated with depth (*p* < 0.01). The abundances of OTU3223 were negatively correlated with temperature, but positively correlated to DO (*p* < 0.01). In contrast, the abundances of OTU1189 were negatively correlated with both temperature and DO (*p* < 0.01). The abundances of OTU1762 (Candidatus *Nitrosopumilus*), OTU3455 (Nitrosopumilaceae) and OTU90 (Woesearchaeia) were all significantly and negatively associated with salinity (*p* < 0.01). The abundances of OTU3233 were significantly and positively correlated with turbidity (*p* < 0.01), while those of OTU1189 were negatively associated with turbidity (*p* < 0.01). The abundances of OTU249 and OTU1433 were significantly and positively correlated with EC and TDS (*p* < 0.01). In contrast to the above, no significant associations were observed between the abundances of dominant OTUs and TN or TP (*p* > 0.05). Lastly, the abundances of OTU932 (Candidatus *Nitrosopumilus*) were notably and negatively correlated with total organic carbon concentrations (TOC, *p* < 0.01).

## Discussion

Microorganisms play important roles in mediating global biogeochemical cycling of essential elements in marine environments [35,36]. Among microbial groups, Archaea are ubiquitously and abundantly distributed in various marine environments, including marine sediments [37,38], coastal waters [39], estuaries [40], and mangrove sediments [41]. Archaea are key mediators of nitrification, sulfur cycling, methane oxidation, and methanogenesis within ocean environments [42–45]. However, the distribution of archaeal diversity and abundances in coastal aquaculture environments and their relationship with external influencing factors remain poorly understood. To fill this knowledge gap, archaeal 16S rRNA gene sequences were generated from communities within an aquaculture ecosystem using high-throughput sequencing methods and then taxonomically classified. The abundance of sequences affiliated with unclassified taxa increased when evaluated from the class to the species level, indicating substantial potential for archaeal biodiversity discovery. Among the communities analysed here, Thaumarchaeota and Nanoarchaeaeota accounted for 83.5% and 13.5% of the total communities, respectively. At a finer taxonomic level, the Nitrososphaeria and Woesearchaeia were the most abundant classes and accounted for 83.5% and 13.5% of the total archaeal communities, respectively.

Thaumarchaeota are ubiquitously distributed among a variety of environments, including soils, sediments, oceans, and freshwaters, and are one of the most abundant archaeal groups involved in environmental ammonia oxidation [46–51]. In particular, species within the Nitrososphaeria class of Thaumarchaeota perform ammonia oxidation, and their discovery has dramatically changed our perception of microbial nitrification and nitrogen cycling [16,17,52]. At a genus level, archaeal communities were dominated by Candidatus *Nitrosopumilus* (Thaumarchaeota) and high relative abundances of *Nitrosopumilus* were also observed in studies on archaeal communities in the Mediterranean Sea and Pacific deep-sea sediments[53,54]. In contrast, Woesearchaeia live in terrestrial environments and exhibit fermentative and symbiotic lifestyles [55]. The sequences assigned to the Nanoarchaeaeota phylum from our samples were all affiliated with the Woesearchaeia. The specific lifestyles exhibited by these organisms could play a significant role in their adaptation to terrestrial and aquatic environments. Interestingly, Thermoplasmata were present in both the sediments and waters analysed here, suggesting their potential role in methanogenic activities in the Qinhuangdao coastal aquaculture zone. In addition, Altiarchaeota and Diapherotrite were only detected in sediments. The uncultivated archaeal group Altiarchaeota are a newly recognized phylum and are potentially one of the most abundant autotrophic taxa within Earth’s crust [56]. In addition, the Diapherotrites have only been detected in a few environments, including forest soils, lagoon sediments, and microbial mats [57]. The Diapherotrites contribute to carbon and hydrogen biogeochemical cycles and likely exhibit symbiotic and/or fermentative-based lifestyles [58]. Taken together, our results indicate that the complex archaeal communities of the Qinhuangdao coastal aquaculture zone might be involved in diverse metabolic processes including carbon and nitrogen cycling.

Microbial populations exhibiting high abundances can exert great influences on the overall structure and ecological function of microbial communities. Therefore, shifts in the relative abundances of predominant microorganisms under different environmental conditions can reflect adaptations of microbial populations to habitat filters. For example, previous research has suggested that ecological niche diversification within benthic microbial communities occurs at the millimetre scale [59–61]. Prior to this study, little was known about the distribution of archaeal populations among sediment and water habitats in the coastal environment of Qinhuangdao. Comparisons of community alpha and beta diversity differentiated communities between the sediments and waters. The two habitat types likely exhibited different archaeal diversities due to the intrinsic features of the ecosystems, which provide specialized niches for the adaptation of unique archaeal populations to either system [62,63]. Our results indicated significant archaeal species (i.e., proxied by OTUs) turnover between habitats. Specifically, the sediment communities generally comprised more OTUs than did those of the waters, suggesting the presence of more complex archaeal communities in the sediments. The greater OTU richness in sediments could be explained by the anoxic conditions of sediments, allowing archaeal growth by fermentation or anaerobic respiration using various electron acceptors [64].

The different habitat types also appeared to significantly influence the abundance of dominant OTUs. For example, the ammonia-oxidizing genus Candidatus *Nitrosopumilus* within the Thaumarchaeota phylum were found in all samples, and especially those from the sediments. These results indicate the possibility of ubiquitous ammonia oxidation in the coastal aquaculture zone, and especially in the sediments. Relatively high abundances of MGII were also observed in the waters, which could be due to their potential roles in metabolising organic matter and other taxa-specific energy requirements [65,66].

There is little information available regarding the occurrence of archaeal populations and environmental parameters. Our results indicate that the influence of geographical and environmental variables on the distribution of dominant taxa should be considered. Latitude has previously been implicated as one of the major factors affecting the distribution of archaeal communities in estuarine ecosystems [67]. Here, some dominant OTUs including OTU3223 and OTU1189 were negatively associated with latitude. Notably, longitude was also positively or negatively associated with the abundances of some dominant OTUs. Previous investigations have indicated that microbial community structures are strongly affected by spatial distances and water depth [68]. For example, Flavobacteria clades exhibit distinct distributional patterns corresponding to variation in depth [69]. Likewise, the influence of water depth was also observed for some dominant archaeal OTUs of our study.

Salinity and temperature are parameters that naturally vary, while nutrient concentrations and DO levels can indicate the extent of anthropogenic activities and their influence [8]. Environmental conditions can determine community composition within specific habitats, and understanding associations between taxa and environmental conditions can elucidate the exact response of individual taxa to environmental changes. Previous studies have indicated that archaeal taxa are less responsive to environmental factors than are bacterial taxa [70]. Our results revealed that some dominant OTUs were significantly associated with important environmental variables, including temperature, DO, salinity, turbidity, EC, TDS, and TOC. Könneke *et al*. [71] reported that *Nitrosopumilus maritimus* was inhibited by organic substrates, even at very low concentrations. Interestingly, OTU932, which was classified as Candidatus *Nitrosopumilus*, was negatively correlated to TOC concentrations, which warrants further investigation. The negative correlations between OTU3223 and OTU1189 with temperature could be explained by their adaptation to cold environments [72]. Nevertheless, most OTUs associated with the Nitrososphaeria, Bathyarchaeia, Thermoplasmata, and Woesearchaeia did not exhibit any clear association with environmental factors and were invariably abundant among water samples. Taken together, these results suggest that the differential responses of archaeal taxa to environmental factors could be explained by distinct properties of individual taxa and because some OTUs could be more sensitive to variation in major environmental parameters.

## Conclusions

This study provides the first description of archaeal diversity and community composition in the sediments and waters of the Qinhuangdao coastal aquaculture zone. Our results predict that the archaeal communities could be involved in nitrogen and carbon cycling activities within the aquaculture zone. Habitat type (i.e., sediments and waters) significantly influenced the distribution of archaeal diversity and abundances of dominant OTUs within the coastal ecosystem. Furthermore, the influence of environmental factors on the distributions of dominant taxa suggested that dominant archaea were sensitive to environmental parameters, which might be due to their distinct lifestyles and biological processes. Additional investigations of functional gene expression and evaluation of the active archaeal populations in the area could further our understanding of the ecological roles of archaea in the sediments and waters of the Qinhuangdao coastal aquaculture zone.
